# Whole genome sequencing of promising *Lactobacillus delbrueckii* subsp. *bulgaricus* strains isolated from Egyptian dairy products for probiotic characteristics

**DOI:** 10.1038/s41598-025-90262-2

**Published:** 2025-02-26

**Authors:** Mostafa F. El-Hosseny, Mervat G. Hassan, M. O. Abdel-Monem, Mohammed G. Seadawy

**Affiliations:** 1 Botany and microbiology department, Faculty of Science, Banha University, Banha, Egypt; 2 Biodefense Center for Infectious and Emerging Diseases, Ministry of Defense, Cairo, Egypt

**Keywords:** Probiotics, *Lactobacilli*, Whole genome sequence, Antimicrobial activity, CRISPR, Nutrition, Microbiology

## Abstract

Probiotics are living microorganisms that, when given in an adequate dose, have a healthy impact on human well-being. With global interest in self-care, dietary supplements especially probiotics is expanding rapidly due to their supported health effects. In this study, a total of twenty-two samples were collected from dairy products in Egypt’s markets, firstly isolated then identified and screened for probiotic properties under stressful conditions as NaCl, acid and bile salt conditions. After evaluation of the antimicrobial effect against widespread gram negative and gram positive human infecting bacteria, besides the antiviral assessment against (*SARS-CoV-2*) virus which has disturbed the world, the antibiotic susceptibility test was done. Only three promising isolates were subjected for whole genome sequence with high-output next generation technology and the obtained data was subjected to a full bioinformatics analysis. The results obtained stated the advance of our isolates to tolerate the stress factors that can struggle in the human gut as well as the antimicrobial effects. All these bright characteristics were confirmed and illustrated in detail through different and reliable genome analysis tools. Our isolates were founded to have stable genome through containing mobile genetic elements like phages and CRISPR clusters that confirm the safety and quality for human health.

**Supplementary Information:**

The online version contains supplementary material available at 10.1038/s41598-025-90262-2.

## Introduction

Probiotics are live bacteria that, when given in the right doses, have a useful impact on human well-being^1^. When a bacterial strain satisfies the criteria for safety, usefulness, and technology, it may be referred to as a probiotic. The safety requirements comprise: Animal or human origin; extraction from healthy individual’s gut; verification of a record of safe usage; and lack of data suggesting a link between the strains and infectious diseases^2^. The strain must fulfill the acid resistance functional parameters, bactericocytes, and bile salts that the gut microbiome releases. Additionally, has the ability to survive, carry out metabolism and growing at the location, resisting pathogens, and colonization within the gut. Genome stability, resistance to bacteriophages, viability and endurance of end products during processing and storage are all factors that contribute to probiotics’ technological usefulness^2^. Due to the good impact on health, probiotics are recommended both before and after antimicrobial therapy to help the body’s regular microbiota return, aid the digestive system, increase nutrient and vitamin absorption, and relieve foot allergies^3,4^. Furthermore, probiotics are used to promote the control of body fat, improvement of lipid components and breakdown of carbohydrates, moreover improving immunity of hosts^5^. Whithin the investigated probiotic microbiota, lactic acid-producing bacteria or LAB are especially prevailing, broadly distributed and common residents of human gut epithelium. Numerous LAB genera are extensively used in fermented dairy products that are significant both traditional and industrial especially *Lactobacillus delbrueckii* subsp. *Bulgaricus*^6^. Gram-positive, non-sporulating, or rod-shaped or cocco-bacilli *L. delbrueckii* bacteria typically have a GC genomic ratio of below fifty percent ^7^. *L. delbrueckii* was utilised as starting organism in the manufacturing of different fermented milk foods, as yoghurt and cheese^8^, and has been overall recognised as safe (GRAS) by the Food and Drug Administration (FDA)^9^. Furthermore, being of dairy origin, generating its dietry formulas would be simple.

This research aimes to inspect a particular *L. delbrueckii* strain of local dairy origin for its probiotic nature through biochemical and molecular analysis which further crowned by whole genome sequence. WGS of this strain will be used as a basis for safety assesment in vitro which can be validated by in vivo testing for human medication in a pharmaceutical form. Genome sequencing provides insights into a species’ biology, aids to detect genes specific for single strain, its transcripts, antimicrobial actions and probiotic characteristics.

## Methodology

### Sampling and isolation

Twenty-two samples were collected from local natural and commercial milk products (cottage-cheese, yogurt and sourmilk) from sporadic Egyptian marketplaces. The collected specimens were then packed, marked, and transferred right away in a polyethylene cooled container to Biodefence Center for infectious and emerging diseases near Almaza in Cairo, Egypt^10^. Before adding 1mL of buffered peptone mixture to the de Man-Rogosa-Sharpe MRS broth (Merck, Germany) as enhancement medium and incubating at 37 °C for 24 h, the curd samples were blended using a stomacher blender in 10 mL of a peptone water buffer and incubated at 37°C for 18 h. 0.1 ml of growing specimens were added to MRS agar (Merck, Germany) plates, which were then anaerobically incubated at 37 °C for 48 h (using anaerobic container and gas bag) for the isolation of *lactobacillus*^11^.

### Microbiological and biochemical identification

On MRS agar plates, clear single positive colonies were sub-cultured three subsequent times to be purified. Each isolated culture was investigated for phenotyping e.g.: form, colour, border and opacity of the colony; and microscopic inspection after Gram staining^12^. Following the manufacturer’s instructions, the VITEK^®^ 2 C system (bioMérieux, France) and the suggested ANC cards were used for accurate identification and biochemical affirmation^13^.

### Evaluation of probiotic characteristics

Recognized as gram-positive, rod-shaped *Lactobacillus* isolates were evaluated for tolerance to sodium chloride, bile salt, acids, antibiotics and ability for inhibiting selected pathogenic microbes.

An inoculum was prepared for each isolate according to the recommended procedure M7-A7-CLSI of Chemical Laboratory Standard Institute^14^ in tubes with 5 ml of broth media and incubated for 24 h at 37 °C. The inoculum’s turbidity was sustained at 0.5 McFarland requirements with 10^7^-10^8^ CFU/ml.

### Tolerance to sodium chloride (NaCl)

With some modification as stated by to Uymaz Tezel et al.^15^, NaCl tolerance was determined. The MRS broth (Merck, Germany) was modified with various NaCl concentrations (0, 3, 4, 5, 6, and 7) (w*/*v) was employed. 1% (v/v) isolate of *Lactobacillus* isolate was inoculated to the freshly made overnight culture in modified MRS and it was then anaerobically incubated at 37ºC for intervals of two to five hours. The trial was conducted in triple manner, with pure MRS broth serving as the control sample. After two and five hours, the absorbance at 600 nm was measured.

### Acid tolerance

Isolates were investigated for viability at acidic medium, as illustrated by Shaikh & Shah^16^. In MRS broth overnighted cultures (1.3 × 10^9^ CFU/ml) were spun down for 10 min at 5000 rpm. Following a two- and five-hour incubation period at 37°C with pellets re-suspended in the PBS buffer modified at pH (3, 4, 5, 6) and pH 7 (control), 1% of each experiment was introduced to fresh MRS broth (pH 7) and kept for 24 h at 37 °C in tripartite. OD_600_ was used to measure the growth. Acid-tolerant isolates were defined as those that exhibited greater than 50% survival at pH 4.

### Bile tolerance

Each isolate were assessed for bile salt tolerance according to the methodology of Vinderola and Reinheimer^17^. Two hundred microliters from each isolated inoculum suspensions (10^7^-10^8^ CFU/ml) were inoculated into 1000 µl of MRS broth containing varying concentrations of bile salt (0.1, 0.2, 0.3, 0.4 and 0.5% w/v) along with a control MRS broth devoid of bile salt, and incubated for 2 and 5 h at 37 C. Subsequent to incubation, the optical density (OD) was assessed at 600 nm for comparison with the control. Isolates exhibiting over 50% resistance at 0.3% bile dose were classified as bile-tolerant one. The tolerance percentage was assessed in both scenarios s (acidic pH / bile levels) as follows:

$$\:\text{\%}\:\text{R}\text{e}\text{s}\text{i}\text{s}\text{t}\text{a}\text{n}\text{c}\text{e}=\frac{Increment\:of\:OD\:in\:MRS\:broth\:with\:bile\:salt/pH3,\:\text{4,5},\:6\:}{Increment\:of\:OD\:in\:MRS\:broth\:with\:bile\:salt/pH\:7}\times\:100$$

### Antibiotics sensitivity

The antibiotic sensitivity of each isolate was assessed using the disc diffusion method on Mueller-Hinton agar (MHA)^18^. The turbidity of the nightly testing inocula (10^7^-10^8^ CFU/ml) was adjusted to 0.5 McFarland. Each isolate was inoculated on MHA with methylene blue (0.5 g/ml) mixed by 5% defibrinated horse blood. BD BBL™ Sensi-Disc™ antibiotic sensitivity discs were positioned on the streaked cultures by sterile forceps; the testing plates were chilled for 2 hours and thereafter incubated for 20 h at 35 °C. Suppression ring diameter was assessed in accordance with the guidelines established by the Laboratory and Clinical Standard Institute^19^.

### Antibacterial activity

Antibacterial efficacy of all isolates towards gram-negative and gram-positive pathogens, including *S. aureus*,* S. typhimurium*,* K. Pneumenia*,* E. coli*,* E. fecalis*,* L.monocytogens* was evaluated on MHA plates by the conventional well diffusion method^20^. Each MHA plate was inoculated with 100 µl of bacterial suspension (10^7^-10^8^ CFU/ml). Each infected plate well was injected with 30 µl *Lactobacillus* cell-free supernatant, derived from centrifugation of 24-hours cultured inoculum in MRS broth for 10 minutes at 4000 rpm. The negative sample employed was MRS broth, wherease the positive control utilized was Ciprofloxacin (30 ug/ml). To ensure that the suppression is not caused by lactic acid instead to the tested isolate, our supernatant broths were adjusted to pH 6.5. All dishes were incubated for 24 h at 37 °C and the inhibition zone was assessed using the automated colony counter Sphere Flash (IUL, Barcelona). Antibacterial effect was observed in isolates exhibiting a suppression zone diameter of 10 mm or greater.

### Antiviral action

The cytotoxicity and plaque-reduction test were done to evaluate the antiviral effect of promising isolates against *SARS-CoV-2* virus (COVID-19). The cytotoxicity effect of the examined isolates on viability and growth of Vero E6 cells were assessed using the 3-(4,5-dimethylthiazol-2-yl)-2, 5-diphenyltetrazolium bromide (MTT) technique, following Mosmann’s guidelines^21^  with minor modifications. Following the preparation of the stockarting concentration of the isolates in 10% DMSO, a serial dilution was performed in Dulbecco’s Modified Eagle’s Medium (DMEM). In summary, 100 µl of bacterial suspension at a concentration of (3 × 10^5^ cells/ml) was inoculated and incubated for 24 h at 37 °C with 5% CO2. Isolate dilutions were applied after 24 h in triplicate. After 24 h, the media was removed, and the cell lines were thoroughly rinsed three times with PBS (phosphate-buffered saline) before being inoculated with 20 µl MTT (5 mg/ml). the cells were then incubated for 4 h at 37 °C, followed by aspiration of the medium. The crystalized formazan was dissolved using 200 µl of acidic isopropanol (0.04 M HCl in pure isopropanol). The absorbance of Formazan mixtures was measured using a suitable scanner at a wavelength 540 nm, with 620 nm serving as the standard wavelength. The formula empolyed to determine the ratio of cytotoxicity relative to untreated cells is as follows:

$$\:\text{\%}\:\text{C}\text{y}\text{t}\text{o}\text{t}\text{o}\text{x}\text{i}\text{c}\text{i}\text{t}\text{y}=\frac{(Absorbance\:of\:untreated\:cells\:-Absorbance\:of\:treated\:cells)}{Absorbance\:of\:untreated\:cells}\times\:100$$

The concentration of a sample that reduces cell viability by 50%, determined by graphing sample concentration against cytotoxicity percentage, is referred to as (CC_50_)^22^.

Plaque reduction experiment was conducted by inoculating a six well-plate with Vero E6 cells (1 × 10^6^ cells/ml) at 37 °C for 24 h in 5% CO2. 100 µl of the isolates’ safe dilutions (10^− 4^, 10^− 5^, 10^− 6^ and 10^− 7^) was combined with (8 × 10^4^ PFU/well) of diluted COVID-19 and incubated at 37 °C for 1 hour prior to inoculation into the cells following the removal of the seeding medium. A 1-hour incubation for viral adsorption was conducted, followed by addition of 3 ml of enriched DMEM containing 2% agarose. Solidified Plates were kept for three days at 37 °C untill viral plaques developed). Following the addition of 10% formalin for 2 h, 0.1% crystal violet was added prior to washing. Positive control was designated for wells infected with the untreated virus. Finally, the percentage reduction in generated plaques relative to the control was determined using the following equation after enumerating the plaques:

$$\:\text{\%}\:Reduction=\frac{(untreated\:viral\:count\:-treated\:viral\:count)}{untreated\:viral\:count}\times\:100$$

A nonlinear regression analysis was conducted using GraphPad Prism^®^ (GraphPad Software Inc., San Diego, CA, USA) version 8 on the percentage decrease in data and the corresponding concentration values to produce sigmoidal dose-response curves, from which the estimated concentration required to achieve a 50% reduction in virus count (IC50) was determined^22^. The selectivity indices, which assess thedisparity between cytotoxicity and antiviral activity^23^ of the investigated isolates, were determined using to the following equation:

$$\:Selectivity\:index=\frac{CC50\:of\:isolate\:on\:control\:Vero\:E6\:cells}{\text{I}\text{C}50\:\text{o}\text{f}\:\text{t}\text{h}\text{e}\:\text{i}\text{s}\text{o}\text{l}\text{a}\text{t}\text{e}\:\text{o}\text{n}\:\text{i}\text{n}\text{f}\text{e}\text{c}\text{t}\text{e}\text{d}\:\text{V}\text{e}\text{r}\text{o}\:\text{E}6\:\text{c}\text{e}\text{l}\text{l}\text{s}}$$

### DNA extraction

Genomic DNA was extracted from the isolates’ broth utilizing the PureLink™ Genomic DNA purification kit (Thermo Fisher Scientific Inc, USA). According to the manufacturer’s instructions, a maximum of (2 × 10^9^) colonies were ollected following centrifugation and subsequently solubilized using Enzymatic Genomic Digestion Buffer. Following the addition of Proteinase K, incubation at 55 °C was conducted untill cell lysis occured, lasting 30 min and 4 h. Subseqently, RNase A was introduced and thoroughly mixed following a 2-minute incubation at room temperature with Genomic Lysis/Binding Buffer. The entire content was thoroughly combined with 100% ethanol and subsequently rinsed twice by buffers AW1 and AW2 respectively. The eluted DNA was quantified for concentration and purity using a calibrated Qubit™ 2.0 fluorometer (Invitrogen, United States) with a DNA HS assay kit and a Nanodrop 8000 (Thermo Fisher Scientific, Waltham, MA, USA) according to the manufacturer’s instructions, and subsequently stored at -20 °C until utilized.

### Genome sequencing

Following the manufacturer’s guidelines, libraries of DNA were prepared using the Nextera-XT DNA Library Prep kit (Illumina, San Diego, CA) with initial DNA concentration of 1 ng/µl. Nextera transposome was employed for fragmentation and tagmentation of tested genomic DNA (gDNA) by incorporating (i7) and (i5) index adapter. Amplified libraries underwent purification with single-sided Agencourt AMPure XP beads, followed by normalization and dilution. According to sample complexity, it’s possible to pool more than three samples per run, which can then be sequenced using the Illumina MiSeq 300-cycle cartridge kit for cluster generation on the Miseq Platform (Illumina, San Diego, CA, United States) at Genome Research Unit, Biodefense Center for infectious and emerging diseases, Ministry of Defense, Egypt.

### Genome features, assembly, annotation

Primary analysis of the sequencing run parameters was done on board after the run had finished. FastQC (version 0.12.1) was utilized on the raw reads^24^. Assembly quality assessment was conducted using the complete genome analysis application available on the PATRIC (PathoSystems Resource Integration Center) software^25^. The acquired data from prior analysis utilizing FastQC and QUAST with satisfactory quality were annotated employing RASTtk (Rapid Annotation using Subsystem Technology tool kit)^26^.

### Phylogenetic tree analysis

Mash/MinHash was used to identify the representative genomes and closest reference^27^. PGFams (PATRIC global protein families) were selected to determine the phylogenetic taxonomy from these genomes^28^. The protein sequences of these families were aligned using MUSCLE (Multiple Sequence Alignment)^29^, and each sequence’s nucleotides were allocated to the protein alignments. Following the concatenating the integrated nucleotide and amino acid alignments into a data matrix, RaxML (Randomized Axelerated Maximum Likelihood)^30^ was employed to efficiently bootstrap this matrix for analysis^31^.

### Bioinformatics analysis

The set of genes associated with probiotic characteristics were identified using (antiSMASH 7.0)^32^. Antibiotic resistance genes were identified by ARTS 2.0 (Antibiotic Resistant Target Seeker)^33^, together with the AMR gene identification tools based on k-mer analysis in PATRIC and ResFinder software (version 4.5.0)^34^. The presumed mobilome and phages, were examined under PhaBOX^35^. The CRISPR (clustered regularly interspaced short palindromic repeats) and CRISPR/Cas (CRISPR associated-genes) were identified using the CRISPRCasFinder^36^.

### Statistical analysis

The standard deviation was alculated for each characterization test, conducted in triplicate. Statistical analysis was conducted using GraphPad Prism 6.0. A p-value below 0.05 was considered statistically noteworthy.

## Results

### Identification of isolates and screening of the probiotic properties

Among twenty-two isolated specimens, 14 samples showed positive identification on MRS selective medium as *Lactobacillus spp*. White, smooth, mucoid colonies have been subjected to microscopic investigation, appeared as gram-positive rod-shaped bacteria. The VITEK® 2 C automated platform provided biochemical confirmation identifying five isolates as *Lactobacillus spp.,* which were further evaluated against specific probiotic characteristics.

### Tolerance to sodium chloride (NaCl)

Out of five *Lactobacillus* isolates was evaluated against different concentrations of NaCl, three tolerant were only well developed at dosages of 3%, 4%, and 5%, but at concentrations of 6% and 7% NaCl slow growth was noticed. At 7% NaCl concentration, growth significantly diminished after both 2 and 5 h of incubation (Table 1).

**Table 1:** Adaptation of *Lactobacillus* isolates for NaCl at 2 and 5 h.

Isolate	NaCl 3%		NaCl 4%		NaCl 5%		NaCl 6%		NaCl 7%	
	count	abs	count	abs	count	abs	count	abs	count	abs
B1-2 h	1326.69	0.728	1736.44	0.873	1234.66	0.622	1145.31	0.581	437.71	0.18
B2-2 h	1400.25	0.73	1596.25	0.881	1145.69	0.56	1326.69	0.67	144.49	0.186
G1-2 h	1261.02	0.834	1011.44	0.69	1387.91	0.795	1068.00	0.564	577.97	0.284
S5-2 h	345.26	0.43	635.35	0.202	510.24	0.221	826.36	0.198	144.02	0.324
S8-2 h	52.54	0.229	78.81	0.266	236.44	0.2	866.95	0.195	65.68	0.303
B1-5 h	1301.23	0.702	1712.36	0.682	1190.25	0.594	1009.13	0.548	359.27	0.201
B2-5 h	1320.89	0.714	1401.36	0.703	1289.25	0.631	1078.51	0.546	328.39	0.138
G1-5 h	1471.19	0.656	1287.49	0.73	1488.96	0.658	1058.75	0.59	144.49	0.11
S5-5 h	354.66	0.202	564.83	0.168	131.36	0.259	775.00	0.193	354.66	0.197
S8-5 h	446.85	0.125	399.75	0.216	762.58	0.179	617.37	0.261	577.97	0.142

### Acid tolerance

Three isolates grown well at 5% concentration of NaCl showed survival also when grown in low pH and results gave a resistance at acid stress. Resistant isolates survive at pH 4 (with tolerance > 50%). Highest tolerance was noticed with G1 (83.19%) and lowest with B1 (71.55%) at pH 4 after 5 h (Table 2). At pH 3, no isolate was discovered to be tolerant (with a resistance percent < 50%).

**Table 2:** Acid tolerance of *Lactobacillus* isolates at 2 and 5 h.

Isolate	pH 3		pH 4		pH 5		pH 6		pH 7		resistance % at pH 4
	count	abs	count	abs	count	abs	count	abs	count	abs	
B1-2 h	131.36	0.478	105.08	0.592	788.14	0.798	507.63	0.658	966.19	0.824	71.84
B2-2 h	92.12	0.359	126.27	0.556	630.51	0.611	605.08	0.712	1050.85	0.811	68.56
G1-2 h	13.14	0.401	39.41	0.552	903.39	0.678	472.88	0.799	828.39	0.876	63.01
S5-2 h	52.54	0.295	13.14	0.205	26.27	0.368	1142.80	0.269	1002.34	0.761	26.94
S8-2 h	266.95	0.29	814.41	0.217	118.22	0.342	170.76	0.281	1172.88	0.868	25
B1-5 h	275.85	0.496	459.75	0.581	1129.66	0.725	964.83	0.723	1170.76	0.812	71.55
B2-5 h	775.00	0.626	39.41	0.711	1090.75	0.756	943.64	0.721	1103.39	0.897	79.26
G1-5 h	205.08	0.587	323.31	0.786	920.34	0.847	880.51	0.716	938.56	0.94	83.19
S5-5 h	52.54	0.659	315.25	0.372	39.41	0.385	78.81	0.423	972.03	0.973	38.23
S8-5 h	144.49	0.651	499.15	0.436	141.53	0.38	551.69	0.428	902.12	0.991	43.99

### Bile tolerance

**Fig. 1:**
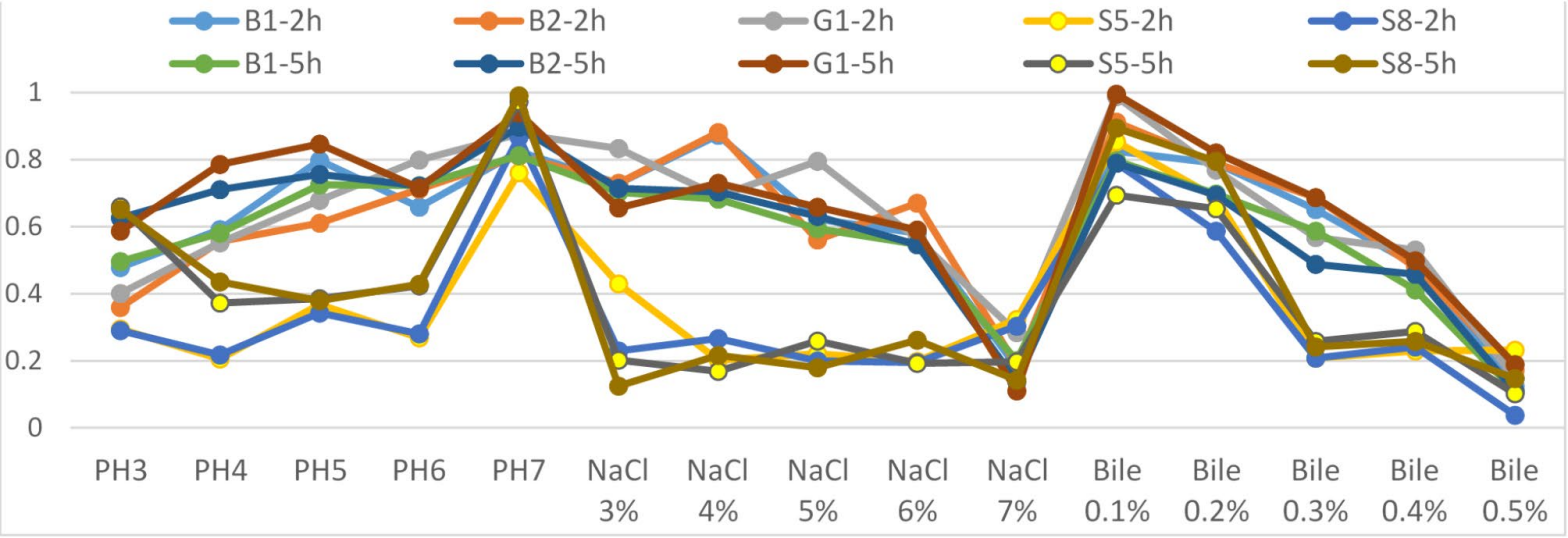
The ability of *Lactobacillus* isolates (B1- B2- G1- S5- S8) to endure varying concentrations of (pH – NaCl - Bile salt) following incubation in MRS broth for 2 and 5 hours at 37 °C.

By increasing the concentration of bile salt, the inhibitory effects were observed, but three tested isolates retained up growing as the incubation period extended (with resistance > 50%), but at a 0.5% concentration of bile salt, it was unable to withstand. Highest resistance was reported with B2 (58.04%) and lowest with G1 (50.15%) at 0.4% after 5 h (Table 3). Despite, no isolate was found tolerant at 0.5% (with percentage resistance < 50%). The challenge of brilliant isolates to withstand at these stress conditions similar to those of the stomach and human gut was illustrated in (Fig. 1).

**Table 3:** Bile resistance of *Lactobacillus* isolates at 2 and 5 h.

Isolate	Bile 0.1%		Bile 0.2%		Bile 0.3%		Bile 0.4%		Bile 0.5%		resistance at 0.4%
	count	abs	count	abs	count	abs	count	abs	count	abs	
B1-2 h	1274.15	0.825	1083.90	0.789	918.75	0.651	309.41	0.486	126.27	0.125	58.9
B2-2 h	713.14	0.912	636.25	0.789	486.02	0.687	96.19	0.478	39.41	0.172	52.41
G1-2 h	998.305	0.988	577.966	0.769	764.830	0.568	236.44	0.531	157.627	0.147	53.74
S5-2 h	144.49	0.854	262.5	0.687	288.14	0.208	249.58	0.229	52.54	0.232	26.81
S8-2 h	26.27	0.789	236.44	0.587	230.51	0.208	398.31	0.241	259.32	0.038	30.54
B1-5 h	1648.75	0.796	1388.75	0.698	1017.5	0.587	1042.5	0.412	131.25	0.109	51.75
B2-5 h	656.25	0.789	546.25	0.694	501.25	0.488	155	0.458	140	0.109	58.04
G1-5 h	935	0.995	867.5	0.82	696.25	0.687	263.75	0.499	128.75	0.189	50.15
S5-5 h	1058.75	0.694	157.5	0.654	570	0.258	254	0.288	210	0.102	41.5
S8-5 h	1125	0.894	262.5	0.795	421.25	0.241	315	0.258	165	0.147	28.86

#### Antibiotics susceptibility

The antibiotic resistance for our trials was investigated on MHA plates by disc diffusion technique and the findings are showed in (Table 4). Three isolates showed resistant to harsh factors, were highly susceptible to each of Ciprofloxacin, Chloramphenicol, azithromycin, Neomycin, Nalidixic acid and Clarithromycin while susceptible to Gentamicin, Streptomycin, Tetracycline, Erythromycin, and Ampicillin. Notably, these isolates exhibit a common less susceptibility against Ceftriaxone, Penicillin and Trimethoprim. It is advantageous for probiotic microbes to exhibit antibiotic-resistant characteristic so as to endure antibiotic therapy in the gastrointestinal system.

**Table 4:** Antibiotic sensitivity results of *L. Delbrueckii* isolates with MHA after 24 hours.

Antibiotics	B1	B2	G1	S5	S8
Ampicillin	++	++	+++	+	R
Azithromycin	++++	++++	++++	++++	+++
Penicillin	+	+	+	++	R
Chloramphenicol	++++	+++	++++	++	+++
Ciprofloxacin	++++	++++	+++	++	+++
Clarithromycin	++++	+++	++++	++++	++++
Erythromycin	++	+++	++	+	R
Tetracycline	++	+	+++	++	R
Nalidixic acid	+++	++++	++++	++++	++++
Trimethoprim	+	+	+	R	++
Ceftriaxone	++	+	+	++	++
Streptomycin	+	++	+++	++	R
Gentamicin	++	++	+	R	+
Neomycin	++++	++++	++++	++++	++++
Sulfamethizole	+	++	++	R	R

R = tolerant, += less sensitive, ++= moderately sensitive, +++= sensitive, ++++= highly sensitive.

### Antibacterial activity

**Fig. 2:**
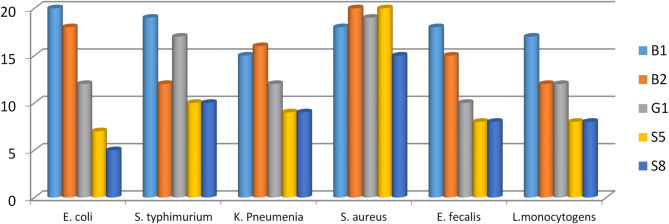
Antibacterial activity of *L. delbrueckii* isolates against pathogenic bacteria.

Using six pathogens, the evaluated isolates’ antibacterial effects were investigated (Fig. 2). Tested bacterial cell-free fluid revealed inhibition action towards the investigated pathogenic bacteria. *S. aureus* was the extreme susceptible pathogen towards all of the investigated isolates. Isolate B1 exhibited the highest potency.

### Antiviral activity

The cytotoxic effect of serial concentrations of examined isolates (B1-B2-G1) on viability of Vero E6 cell line for 24 hours, showed that (10^− 4^, 10^− 5^, 10^− 6^ and 10^− 7^) concentrations was safe and these concentrations were investigated for antiviral effect towards (covid-19). The results indicated an inhibition % as presented in (Table 5). Isolate B1 was identified as the active inhibitor.

**Table 5:** Antiviral effect of *L. Delbrueckii* isolates on *SARS-CoV-2*.

Sample	Conc (CFU/ml)	Cytotoxicity %	Initial Viral count (PFU/ml)	Post Viral count (PFU/ml)	Inhibition %
B1	10^− 4^	73.12	8*10^4^	2.8*10^4^	65
	10^− 5^	67.19		5.2*10^4^	35
	10^− 6^	52.62		5.52*10^4^	31
	10^− 7^	45.34		7.76*10^4^	3
B2	10^− 4^	70.59	8*10^4^	3.2*10^4^	60
	10^− 5^	59.32		4.48*10^4^	44
	10^− 6^	53.38		5.52*10^4^	31
	10^− 7^	48.92		6.48*10^4^	19
G1	10^− 4^	67.48	8*10^4^	3*10^4^	62
	10^− 5^	63.26		5.11*10^4^	36
	10^− 6^	51.17		5.99*10^4^	25
	10^− 7^	51.15		7.12*10^4^	11

### Genome sequencing

We acquired a comprehensive genome sequencing of *Lactobacillus* strains. The sequencing run was conducted on Miseq machine for three potential samples discovered during the investigation by Nextera XT library preparation kit. The analyzed data yielded a total of 1350 k/mm2 clusters, with 90.8% classified as cluster PF, 96.5% Q30 for Read 1 and 97.5% Q30 for Read 2.

### Genome features, assembly, annotation

The raw reads exhibited a favorable quality score during FastQC quality control assessments of the raw sequencing data. The results indicates that sample no.1, designated as (B1), yielded 639 contigs and a genome size of 3,954,441 bp, With a G + C composition of 44.20%. The N50 length, representing the shortest sequencing fragment at 50% of the genome, is 28,871 bp. The minimum number of contigs required to achieve a N50, which determines the L50 count, is 32. Sample (B1) was annotated using RASTtk (RAST tool kit) and assigned a specific taxonomic category as follows: Cellular organisms > Bacteria > Terrabacteria > Firmicutes > Bacilli > Lactobacillales > Lactobacillaceae. This genome has 4,691 protein coding sequences (CDS), 100 transfer RNA (tRNA) genes, and 7 ribosomal RNA (rRNA) genes. The annotation encompssed 1,339 hypothetical proteins and 3,352 proteins with functional assignments, including 1,156 EC numbers (proteins with Enzyme Commission designations), 981 GO assignments (proteins with Gene Ontology classifications), and 820 proteins mapped to KEGG pathways^37,38^ as well as 4,420 proteins classified within PGFams (cross-genus protein families).

**Table 6:** The genomic features of the sequenced isolates’ assembly and annotation.

Isolate (Job ID)	B1 (2699059)	B2 (898488)	G1 (3155671)
Source	yogurt	yogurt	sour milk
Assembly details:			
Contigs	639	1022	929
GC Content	44.20	49.14	46.29
Contig L50	32	91	69
Genome Length (bp)	3,954,441	2,136,850	5,499,788
Contig N50	28,871	5,801	18,019
Total genes	3795	1964	5353
Core/ Essential genes	747	537	806
Annotated Genome Features:			
CDS	4,691	2,896	6,422
Repeat Regions	247	151	345
tRNA	100	62	139
rRNA	7	4	15
Protein Features:			
Hypothetical proteins	1,339	906	1,867
Proteins with functional assignments	3,352	1,990	4,555
Proteins with EC number assignments	1,156	651	1,600
Proteins with GO assignments	981	543	1,355
Proteins with Pathway assignments	820	446	1,134
Proteins with PATRIC (PGfam)	4,420	2,691	6,051

The genomic characteristics of the sequenced samples, designated as B1, B2 and G1 are detailed in Table 6. TheSamples labeled by B2 and G1 yielded 1022 and 929 contigs, respectively, with genomic lengths of 2,136,850 and 5,499,788 bp, exhibiting atypical GC content of 49.14% and 46.29%. The N50 length is 5,801 and 18,019 bp, with L50 count of 91 and 69, respectively. The annotation indicates that both genomes belong to the Lactobacillaceae family. The genomes contain 2,896 and 6,422 coding sequences (CDS), 4 and 15 rRNA genes, and 62 and 139 tRNA genes. The annotation includes 906 and 1,867 hypothetical proteins, and 1,990 and 4,555 proteins with functional assignments, categorized as 651 and 1,660 EC proteins, 543 and 1,355 with GO assignments, and 446 and 1,134 proteins mapped to KEGG pathways^37,38^ along with 2,691 and 6,051 PGFams proteins.

**Fig. 3:**
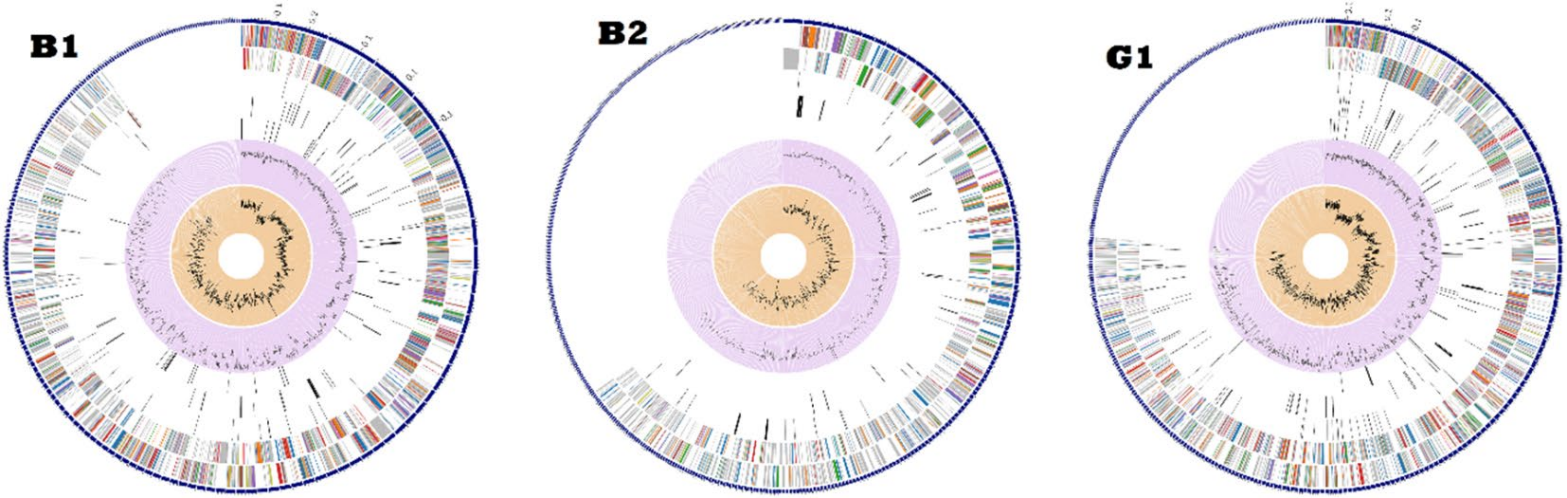
The circular graph of the contigs distribution throughout the genome mapped annotations.

**Fig. 4:**
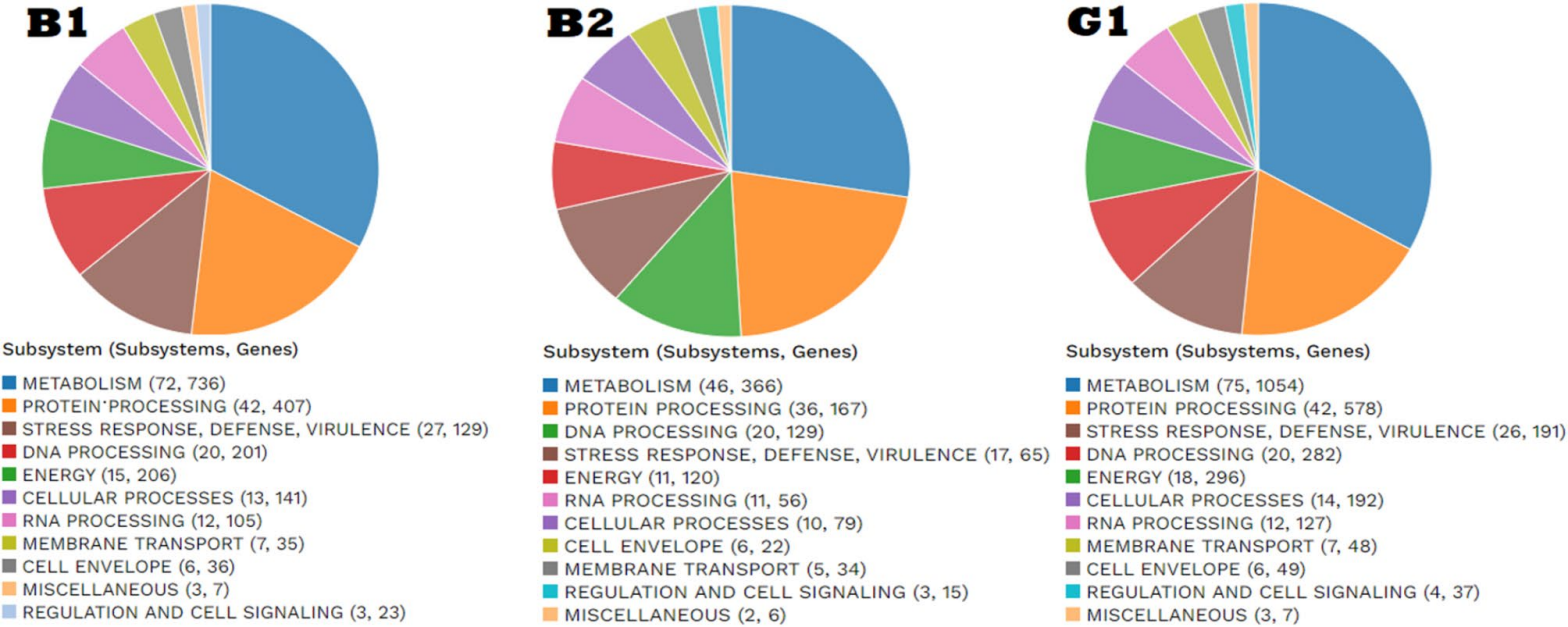
The circular diagram representation of the subsystem (subsystems, genes).

The allocation of the genomic annotations is illustrated in a circled graph in (Fig. 3). This includes, from outermost to inner rings, contigs, CDS on the forward strand, CDS on the reverse strand, RNA genes, CDS homologous to recognized antimicrobial-resistance genes, CDS homologous to known factors of virulence, GC content and GC skew. The subsystem associated with these genes is denoted by the colors of the CDS on both the forward and reverse strands (Fig. 4).

The subsystem is an assembly of proteins that collaboratively perform a certain biological task or structural complex. The PATRIC annotation procedure includes an analysis of the subsystems specific to each genome. (Fig. 4) summarizes the subsystem contents for these genomes. The predominant features of the subsystem include metabolic processes, Protein processing, Defense, reaction to Stress, Energy, Virulence, Cellular activities, DNA processing, RNA production, Cell outer membrane, Membrane transport, Miscellaneous operations, Regulation, and Cell signaling.

### Phylogenetic tree analysis

**Fig. 5:**
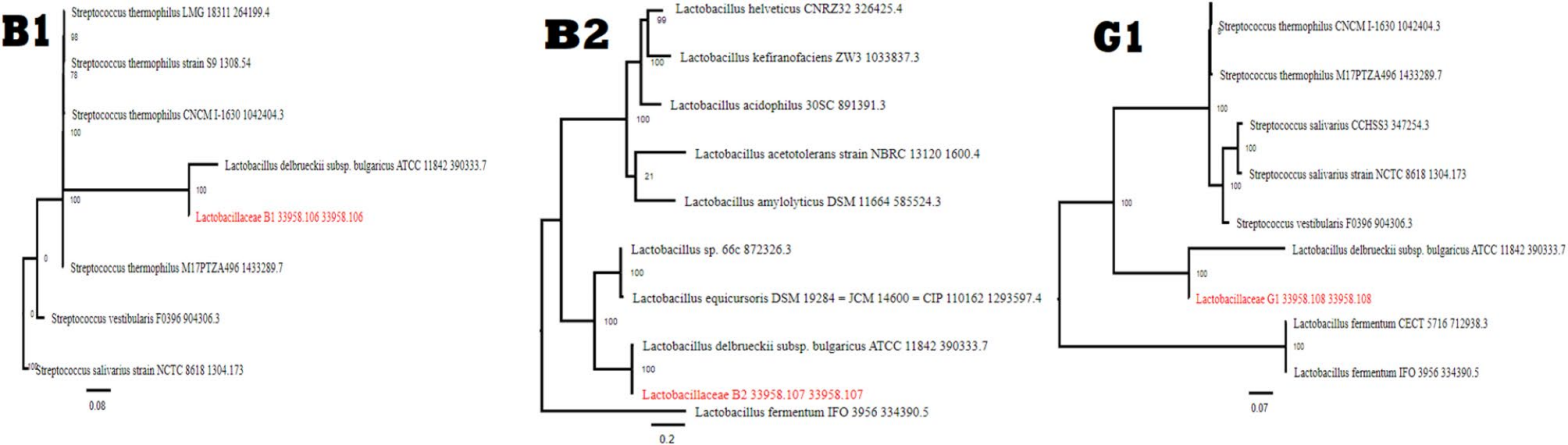
The phylogenetic tree illustrates the predominant strain to which our isolates are classified in the adjacent taxonomy.

The three strains exhibit highest sequence matches with *Lactobacillus delbrueckii* subsp. *Bulgaricus.* A phylogenetic tree was constructed from the complete genomic sequence by concatenating nucleotide and amino acid alignments into a data matrix, which was then analyzed using RaxML to elucidate the phylogenetic relationship among *Lactobacillus* strains as illustrated in (Fig. 5).

### Bioinformatics analysis

A multitude of annotated genes exhibit homology to established drugs targets, transporters, and antibiotic resistant genes. The presence of AMR-related genes in a genome does not necessarily indicate an antibiotic resistance phenotype, as proven by ResFinder software (version 4.5.0), which demonstrated that the samples exhibited no resistant phenotypes. It is essential to investigate specific AMR pathways, including the presence or absence of SNP mutations that signify resistance. Table 7 presents a summary of the AMR genes found in this genome, along with the corresponding AMR pathway.

**Table 7:** Genes related to specialty and AMR in the sequenced isolates.

Strain (Job ID)	B1 (2699059)	B2 (898488)	G1 (3155671)
Specialty Genes:			
Antibiotic Resistance	45	22	67
Drug Target	11	5	12
Transporter	34	8	36
Antimicrobial Resistance Genes:			
Antibiotic target in susceptible species	Alr, Ddl, EF-G, EF-Tu, folA, Dfr, folP, gyrA, gyrB, inhA, fabI, Iso-tRNA, kasA, MurA, rpoB, rpoC, S10p, S12p	Alr, Ddl, EF-G, EF-Tu, folA, Dfr, folP, gyrA, gyrB, inhA, fabI, Iso-tRNA, kasA, MurA, rpoB, rpoC, S10p, S12p	Alr, Ddl, EF-G, EF-Tu, folA, Dfr, folP, gyrA, gyrB, inhA, fabI, Iso-tRNA, kasA, MurA, rpoB, rpoC, S10p, S12p
Antibiotic target modifying enzyme	RlmA(II)	--	RlmA(II)
Antibiotic target replacement protein	FabK	--	FabK
Gene conferring resistance via absence	gidB	gidB	gidB
Protein altering cell wall charge conferring antibiotic resistance	GdpD, MprF, PgsA	GdpD, PgsA	GdpD, MprF, PgsA
Regulator modulating expression of antibiotic resistance genes	LiaF, LiaR, LiaS	--	LiaF, LiaR, LiaS

**Fig. 6:**
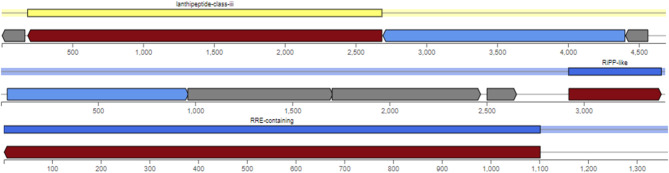
A graphical representation of the secondary metabolite’s abundance across the genome of B2 isolate.

AntiSMASH facilitates the rapid identification, annotation, and analysis of clusters of gene associateded with secondary metabolite synthesis across the entire bacterial genome. It revealed that regions responsible for secondary metabolites, particularly biosynthetic genes, are located on various contigs within the genomes of the examined isolates, as illustrated in (Table 8). The genomic distribution of secondary metabolites in the B2 isolate is depicted in (Fig. 6).

**Table 8:** Biosynthetic secondary metabolites monitored by antismash software.

	B1	B2	G1
RRE-containing	contig_1 - Region 1	contig_326 - Region 1	contig_2 - Region 1
	contig_405 - Region 1		contig_525 - Region 1
RiPP-like	contig_66 - Region 1	contig_165 - Region 1	contig_5 - Region 1
	contig_216 - Region 1		contig_124 - Region 1
	contig_269 - Region 1		contig_317 - Region 1
			contig_425 - Region 1
NRPS-like	contig_127 - Region 1		contig_6 - Region 1
RaS-RiPP	contig_1 - Region 2		contig_16 - Region 1
	contig_14 - Region 1		contig_28-Region 1
lanthipeptide-class-i	contig_111 - Region 1		contig_8 - Region 1
lanthipeptide-class-iii	contig_169 - Region 1	contig_115 - Region 1	contig_263-Region 1
lanthipeptide-class-iv	contig_3 - Region 1		contig_71-Region 1

The ARTS software verified all the resulted analysis derived from AntiSMASH concerning core/essential genes in addition to bacteriocin responsible gene on the contig_66 and contig_269 for (B1) isolate, no more hits for (B2) and bacteriocin localized hits on the contig_5, contig_124 and contig_425 for (G1) isolate.

**Table 9:** Phage-detected contigs in (B1) isolate using PhaBOX.

ID	Contig no.	Length	Lifestyle	Family	Host	Type
1	assembly_contig_22	37,288	virulent	unknown	*Lactobacillus delbrueckii*	CRISPR
2	assembly_contig_37	20,789	temperate	Peduoviridae	*Prevotella nigrescens*	CRISPR
3	assembly_contig_73	12,489	temperate	unknown	*Bacteroides fragilis*	CRISPR
4	assembly_contig_88	9906	virulent	unknown	*Acinetobacter radioresistens*	CRISPR
5	assembly_contig_97	8711	virulent	Herelleviridae	*Lactobacillus delbrueckii*	CRISPR
6	assembly_contig_151	5150	virulent	unknown	*Flavobacterium psychrophilum*	CRISPR
7	assembly_contig_158	4879	virulent	unknown	*Streptomyces sp. SM12*	CRISPR
8	assembly_contig_163	4777	virulent	unknown	*Limnohabitans sp. Jir72*	CRISPR
9	assembly_contig_168	4620	virulent	Ackermannviridae	*Methanobrevibacter smithii*	CRISPR
10	assembly_contig_208	3572	temperate	Ackermannviridae	*Clostridioides difficile*	CRISPR

**Fig. 7:**
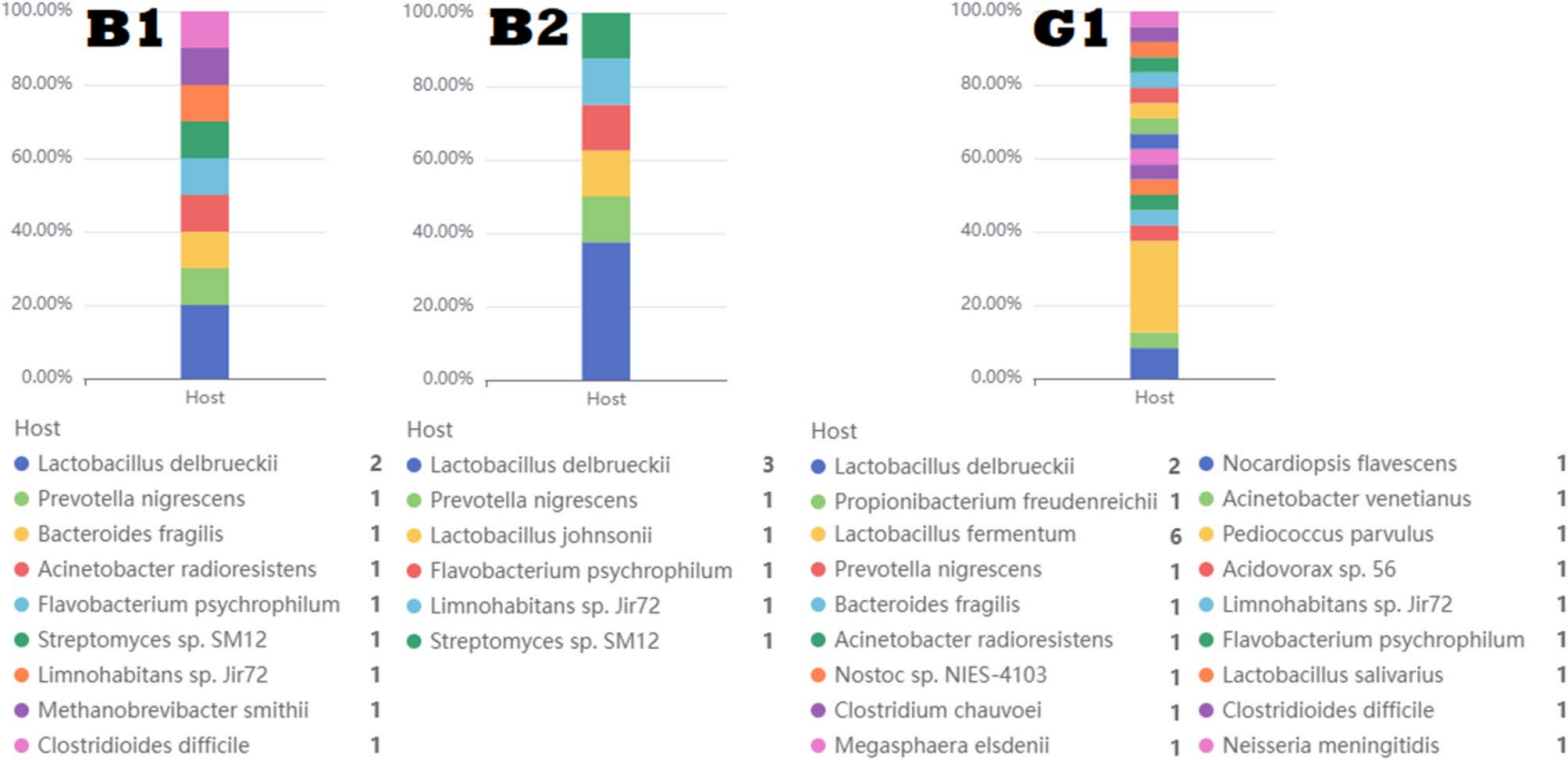
A bar graph depicting the prevalence ratio of various phages througout our samples (B1, B2, G1).

**Fig. 8:**
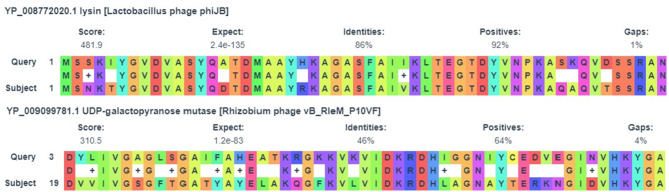
An alignment of some phage segments of (B1) isolate to identify the appropriate host.

Mobile genetic elements, such as prophages and other insertion sequences were detected in the studied isolates by PhaBOX online software. All isolates were determined to have phage sequences either virulent or temperate. The relative abundance of various phages in the examined samples (B1, B2, G1) was illustrated in (Fig. 7). Isolate (B1) has ten phage sequences out of which, three phages belong to (Peduoviridae, Herelleviridae, Ackermannviridae) families that correlated with several appropriate hosts as *L. delbrueckii* as cleared in (Fig. 8). Isolate (B2) showed eight phages, three of which belong to (Peduoviridae, Zierdtviridae, Herelleviridae). Isolate (G1) has the largest number (24) phages, six phages belong to the same families like isolate (B1) plus Casjensviridae family. The remaining identified phages belong to unknown families but correspond to the appropriate host as showed in (Tables 9, 10 and 11).

**Table 10:** Phage detected contigs in (B2) isolate by PhaBOX.

ID	Contig no.	Length	Lifestyle	Family	Host	Type
1	assembly_contig_1	37,325	virulent	unknown	*Lactobacillus delbrueckii*	CRISPR
2	assembly_contig_25	13,359	virulent	Peduoviridae	*Prevotella nigrescens*	CRISPR
3	assembly_contig_61	7753	virulent	Zierdtviridae	*Lactobacillus johnsonii*	Predict
4	assembly_contig_91	5689	virulent	unknown	*Flavobacterium psychrophilum*	CRISPR
5	assembly_contig_108	4834	virulent	unknown	*Limnohabitans sp. Jir72*	CRISPR
6	assembly_contig_117	4553	virulent	Herelleviridae	*Lactobacillus delbrueckii*	CRISPR
7	assembly_contig_143	3864	virulent	unknown	*Streptomyces sp. SM12*	CRISPR
8	assembly_contig_147	3704	virulent	unknown	*Lactobacillus delbrueckii*	CRISPR

**Table 11:** Phage detected contigs in (G1) isolate by PhaBOX.

ID	Contig no.	Length	Lifestyle	Family	Host	Type
1	assembly_contig_24	37,288	virulent	unknown	*Lactobacillus delbrueckii*	CRISPR
2	assembly_contig_36	27,882	temperate	Casjensviridae	*Propionibacterium freudenreichii*	CRISPR
3	assembly_contig_42	24,811	temperate	unknown	*Lactobacillus fermentum*	CRISPR
4	assembly_contig_88	13,546	virulent	Peduoviridae	*Prevotella nigrescens*	CRISPR
5	assembly_contig_90	13,479	virulent	unknown	*Lactobacillus fermentum*	CRISPR
6	assembly_contig_102	12,400	temperate	unknown	*Bacteroides fragilis*	CRISPR
7	assembly_contig_115	10,884	temperate	unknown	*Lactobacillus fermentum*	CRISPR
8	assembly_contig_127	10,046	virulent	unknown	*Acinetobacter radioresistens*	CRISPR
9	assembly_contig_143	8625	temperate	unknown	*Lactobacillus fermentum*	CRISPR
10	assembly_contig_162	7514	temperate	unknown	*Nostoc sp. NIES-4103*	CRISPR
11	assembly_contig_168	7385	virulent	unknown	*Clostridium chauvoei*	CRISPR
12	assembly_contig_182	7155	temperate	unknown	*Lactobacillus fermentum*	CRISPR
13	assembly_contig_183	7060	virulent	unknown	*Megasphaera elsdenii*	CRISPR
14	assembly_contig_196	6624	virulent	unknown	*Nocardiopsis flavescens*	CRISPR
15	assembly_contig_204	6339	virulent	unknown	*Acinetobacter venetianus*	CRISPR
16	assembly_contig_212	6106	temperate	Herelleviridae	*Pediococcus parvulus*	CRISPR
17	assembly_contig_213	6106	virulent	Herelleviridae	*Acidovorax sp. 56*	CRISPR
18	assembly_contig_224	5452	virulent	unknown	*Limnohabitans sp. Jir72*	CRISPR
19	assembly_contig_240	5150	virulent	unknown	*Flavobacterium psychrophilum*	CRISPR
20	assembly_contig_258	4742	virulent	Herelleviridae	*Lactobacillus delbrueckii*	CRISPR
21	assembly_contig_285	4093	temperate	unknown	*Lactobacillus salivarius*	CRISPR
22	assembly_contig_309	3572	virulent	Ackermannviridae	*Clostridioides difficile*	CRISPR
23	assembly_contig_318	3389	temperate	unknown	*Lactobacillus fermentum*	CRISPR
24	assembly_contig_323	3318	virulent	unknown	*Neisseria meningitidis*	CRISPR

Each isolate were examined for anticipated CRISPR-Cas coding sequences. All three isolates exhibited repetitive sequences that encompass one or more related Cas genes or consensus repeats, together with the spacer genes as seen in the subsequent tables. They possess (12, 4, 23) CRISPR sequences, respectively, correlated with (9, 4, 10) Cas in the same sequence and distinguished to (28, 12, 31) clusters in the arrange (B1, B2, G1) as in (Tables 12, 13 and 14).

**Table 12:** CRISPR/Cas clusters detected in (B1) isolate by CRISPRCasFinder software.

Genome location	Length (bp)	CRISPR	Spacer	Cas	genes	Cas clusters
contig_1	140,624	3	19	2	3	Cas2_0_I-II-III, Cas9_0_II, Cas1_0_II
contig_25	32,898	1	18	2	4	Cas9_1_II, Csn2_0_IIA, Cas1_0_II, Cas2_0_I-II-III
contig_45	18,684	1	4	1	9	Cas10_0_IIIA, Cas1_0_I-II-III, Cas2_0_I-II-III-V, Cas6_0_I-III, Csm2_0_IIIA, Csm3_0_IIIA, Csm4_0_IIIA, Csm5_0_IIIA, Csm6_0_IIIA
contig_53	15,742	1	21	2	8	Cas10_0_IIIA, Cas2_0_I-II-III, Cas6_0_I-III, Csm2_0_IIIA, Csm3_0_IIIA, Csm3_0_IIID, Csm4_0_IIIA, Cas1_0_II
contig_110	7658	1	15	2	4	Cas9_0_II, Csn2_0_IIA, Cas1_0_II, Cas2_0_I-II-III
contig_201	3728	1	60	0	0	
contig_261	2468	1	36	0	0	
contig_303	1890	1	29	0	0	
contig_324	1679	1	24	0	0	
contig_452	886	1	13	0	0	

**Table 13:** CRISPR/Cas clusters detected in (B2) isolate by CRISPRCasFinder software.

Genome location	Length (bp)	CRISPR	Spacer	Cas	genes	Cas clusters
contig_17	15,882	1	21	2	8	Cas10_0_IIIA, Cas2_0_I-II-III, Cas6_0_I-III, Csm2_0_IIIA, Csm3_0_IIIA, Csm3_0_IIID, Csm4_0_IIIA, Cas1_0_II
contig_68	7141	1	6	2	4	Cas9_0_II, Csn2_0_IIA, Cas1_0_II, Cas2_0_I-II-III
contig_122	4265	1	62	0	0	
contig_141	3903	1	56	0	0	

**Table 14:** CRISPR/Cas clusters detected in (G1) isolate by CRISPRCasFinder software.

Genome location	Length (bp)	CRISPR	Spacer	Cas	genes	Cas clusters
contig_1	152,846	8	114	1	2	Cas3_0_I, Cmr5_0_IIIC
contig_5	99,259	1	3	1	3	Cas1_0_I-II-III, Cas2_0_I-II-III-V, Csm6_0_IIIA
contig_65	17,874	1	25	0	0	
contig_76	15,825	1	21	2	8	Cas10_0_IIIA, Cas2_0_I-II-III, Cas6_0_I-III, Csm2_0_IIIA, Csm3_0_IIIA, Csm3_0_IIID, Csm4_0_IIIA, Cas1_0_II
contig_90	13,479	1	1	0	0	
contig_132	9369	1	1	0	0	
contig_146	8334	1	50	2	3	Csn2_0_IIA, Cas1_0_II, Cas2_0_I-II-III
contig_160	7709	1	15	2	4	Cas9_0_II, Csn2_0_IIA, Cas1_0_II, Cas2_0_I-II-III
contig_171	7270	1	40	1	5	Cas1_0_IE, Cas2_0_IE, Cas5_0_IE, Cas6_0_IE, Cas7_0_IE
contig_185	6982	1	4	1	6	Cas10_0_IIIA, Cas6_0_I-III, Csm2_0_IIIA, Csm3_0_IIIA, Csm4_0_IIIA, Csm5_0_IIIA
contig_296	3812	1	61	0	0	
contig_396	2142	1	34	0	0	
contig_449	1678	1	24	0	0	
contig_507	1220	1	17	0	0	
contig_542	1022	1	14	0	0	
contig_555	970	1	14	0	0	

## Discussion

The World Health Organization specifies probiotics as “living microbes that, when administered in proper quantities, convey health advantages to the host.” Numerous food products on the market currently contain live microorganisms that are intentionally added with promised general health advantages. Probiotics were employed in a wide range of products globally. Probiotics have been employed in medicines, animal feed, and foods. Since all probiotics used in the dietary supplements available in the Egyptian medicine market are imported from abroad, the need arose to discover and characterize a local strain that is promising as a probiotic prescribed in foods and medicines. The parameters utilized for in vitro choice of probiotics comprise acid and bile tolerance, which allows them to persist and flourish in the GIT (gastrointestinal tract)^39^.

In our study, twenty-two samples were collected from local natural and commercial milk products from different Egyptian markets was cultivated using MRS agar. Only five isolates, B1, B2, G1, S5 and S8 were confirmed as *Lactobacillus spp.* With 99% excellent confirmation and underwent probiotic properties characterization. Out of these 5 isolates, 3 isolates, B1, B2, G1 exhibited high tolerance to 5% NaCl after 5 h and survive well at pH 4 (with tolerance > 50%). Highest tolerance was noticed in G1 (83.19%) and lowest with B1 (71.55%) at pH 4 after 5 h (Table 2). But no isolate (with tolerance percent < 50%) was found to be tolerant at pH 3. For strains to withstand initial stress in the stomach (pH 2–3), persistence in acidic pH is critical^11,40^.

Idoui^41^, demonstrated the persistence of *L. plantarum BJ0021* at 3.0 pH and demonstrated that, in contrast to isolated *Lactobacillus* from human gastro tracts, strains of *L. fermentum*,* L. delbrueckii* subsp. *bulgaricus*, and *L. gasseri* had greater acid tolerance. Additionally, acid tolerant isolates developed and endured at a bile concentration of 0.4% lasting 5 h. Highly resistant one was B2 (58.04%) and lowest was G1 (50.15%) at 0.4% after 5 h (Table 3), while no isolate could resist at 0.5% (resistance < 50%) this was compatible with the evidence of the probiotic isolates’ resistance to pH 3 and 0.3% bile^16^. According to Gilliland^42^, isolated *lactobacilli* from animals guts showed great resistance to bile salts compared to that isolated from dairy goods. Patel et al. obtained similar results^43^. Gilliland et al.^44^ founded that, *L. acidophilus* strains isolated from bovine intestines vary greatly in their capacity to persist in vitro in the bile salt environment. Garriga et al.^45^ demonstrated that chosen LAB strains exhibit tolerance to 4% bile salt. *Lactobacilli’s* resistance varies because of the presence hydrolase -bile salt enzyme- which conjugate bile to lessen toxic effects^46^.

Antibiotic sensitivity can act as appropriate parameter for probiotic culture choice^47^. The sensitivity of all examined isolates to various antibiotics was detected using the agar disc diffusion technique. The selected isolates demonstrated resistance to several antibiotics, in line with Botes et al.^48^ who found that *L. casei* was suppressed by numerous retail antibiotics. *Lactobacilli* are susceptible to penicillin and β-lactamase that target cell wall but resistant to cephalosporins^49^. Most inhibitors against nucleic acid synthesis appear to have less inhibiting impact in a bulk of *Lactobacillus* strains. *Lactobacilli*, however, were often susceptible to low oncentrations of various protein synthesis inhibitors, such as tetracycline and chloramphenicol, due to the presence of its resistance genes, which were sometimes identified in conjunction with others^50^. While Antibiotic resistance in LAB facilitates survival in the gastrointestinal tract during antibiotic treatment, our resistant isolates to stressors exhibited significant sensitivity to Neomycin, Clarithromycin, Ciprofloxacin, Chloramphenicol, Nalidixic acid and azithromycin while sensitive to Gentamicin, Streptomycin, Tetracycline, Erythromycin, and Ampicillin. Notably, these isolates have reduced sensitivity to Ceftriaxone, Trimethoprim and Penicillin.

Because probiotic bacterium play a vital function in people well-being by controlling pathogenic species in the digestive tract and strengthening immunity^51,52^, antibacterial action is among probiotics’ primary characteristics. Consequently, the selected isolates were evaluated towards six pathogens as indicated in (Fig. 2). All bacterial supernatants revealed inhibition towards the pathogens under test. *S. aureus* was the greatest susceptible pathogen against all tested supernatants. Isolate B1 was the most potent isolate. Tulumoglu et al. examined the antibacterial effectiveness of probiotics on *S. aureus*, *P. aeruginosa*, and *E. coli*^47^. Probiotic strains’ antibacterial activity could be related to pH values, competition for resources, or the production of chemicals exhibiting bacteriostatic or bactericidal properties, such as bacteriocin and bacteriocin-like substances ^53,54^. Antimicrobial metabolites, such as acetic acid, lactic acid, carbon dioxide, diacetyl, aldehydes, hydrogen peroxide and other compounds that inhibit pathogenic microorganisms^55^.

As expected, the successful antimicrobial isolates exhibiting antiviral activity toward *SARS-CoV-2* after safe viability testing on Vero E6 cell-line for 24 h. The results in (Table 5) showed an inhibition percentage for B1 isolate which was the active inhibitory one.

The promising isolates B1, B2, G1 exhibiting resistance against acid pH, bile salt and some antibiotics was selected for further study after they also showed antibacterial and antiviral action. All isolates of a milk origin and its various probiotic qualities were studied and examined for effectiveness and safety in human usage. All isolates underwent high-throughput whole-genome next-generation sequencing and were examined for functional characterization. Although this technique is still costly, the charge related to WGS technology has been dramatically decreasing within the last ten years and proceeds to decline with the debut of next-generation systems^56^.

Next-generation sequencing of DNA has quickly become a comprehensive approach for identifying and analysing materials. The raw data resulted by WGS was analyzed through a various pipelines mentioned in methodology. This technique can be adjusted to the necessary taxonomic precision needed, such as the identification of a microbe at the strain scale^57^. In general, high-throughput sequencing offered incredible data that summarised the value of examined strains. WGS succeeded for B1, B2, G1 isolates with superior parameters arose from onboard primary analysis, yielding 1350 k/mm2 total clusters, 90.8% cluster PF and 97% total Q30. Subsequently, the raw reads were subjected to additional evaluation, resulting in a commendable quality score on the FastQC tool.

All reads were assembled and annotated revealing (639 contigs with 3.9 Mbp genome length, 44.20% GC content) for B1 isolate, (1022 contigs with 2.1 Mbp genome length, 49.14% GC content) for B2 isolate and (929 contigs with 5.5 Mbp genome length, 46.29% GC content) for G1 isolate. Omic-based techniques and genetic sequencing are effective approaches to determine possible probiotic-linked responsible genes related to metabolism, genomic adaptability and stability, discovery of antimicrobial tolerance phenotypes, virulence indicators and safety. In addition, in vivo, in vitro, and omic research have been utilised to detect the appropriate bacteria and its related probiotic abilities, including tolerance to acidic environments, bile acids, antimicrobial substances, immuno-modulation, and adhesive mechanisms^58^. Probiotics confer health advantages to their recipients by modifying the gut microbiota, therefore limiting pathogen colonisation, regulating the host immunological reacting, reducing serum cholesterol, exhibiting antidiabetic, antihypertensive and antioxidant actions, and creating bacteriocins^59^. All three isolates have a tight relationship with *Lactobacillus delbrueckii* subsp. *Bulgaricus.* Phylogenetic tree was constructed following the integration of nucleotide and protein alignments into a concatenated matrix and the data were analyzed using PATRIC and RaxML as a section of extensive genomic study. All this data was submitted to the NCBI database.

The values of these genome subsystems are provided in (Fig. 4) that most represented by metabolic processes, Protein processing, Defense, reaction to stress, Energy, Virulence, cellular activities, DNA processing, RNA production, cell outer membrane, Membrane transport, Miscellaneous operations, Regulation and Cell signaling. In our research, the genome was studied for the screening of antimicrobial tolerance, mobile genetic elements as prophages and CRISPR/Cas system. Antibiotic resistance is a worry as the microbiota may transmit antibiotic-resistant genes to pathogenic microbes^60^. Employing the web-based bioinformatics application ResFinder and ARTS, the genomic study revealed that, no antibiotic resistance-related phenotypes or genotypes were found. *Lactobacilli* is recognized for their antibiotic susceptibility based on in vitro research^61^. This finding was most likely influenced by the environment or stress that the bacteria were exposed to. The resulting analysis of ARTS software related to core/essential genes verified all resulted from AntiSMASH.

Our work discovered prophage sequences by PhaBOX online tool that was either virulent or temperate. Most identified phages belong to (Peduoviridae, Herelleviridae, Ackermannviridae, Zierdtviridae and Casjensviridae) families that correlated with certain appropriate hosts as *L. delbrueckii.*

Prophages, on the other hand, participate in the host’s genome elasticity by regulating the individual’s development and viability in the gastrointestinal tract while simultaneously protecting host cells against invasive pathogenic bacteriophages and antimicrobial agents^62^.

As shown in (Tables 11, 12 and 13), our isolates have CRISPR clusters in their genomes along with Cas-gene and spacer. CRISPR clusters inside a genome inhibit the dissemination of antimicrobial resistance genes by obstructing several processes of horizontal gene transfer^63^. The presence of effective CRISPR segments offers a strain a sequence-specific defense mechanism against phages, plasmids and insertion sequences. The CRISPR loci, together with associated Cas genes, provide onto the host strain the capability to defend against invading extra-chromosomal genetic entities^64^. This indicates the stability of our analyzed genomes and a small probability of the strain gaining antimicrobial resistance genes, as resistant genes are often disseminated by mobile genetic components.

CRISPR-Cas systems, while offering a potent adaptive immune defense against mobile genetic elements (MGEs), are subject to several limitations that can compromise their protective efficacy. These limitations include the emergence of escape mutations within the protospacer region of MGEs, disrupting target recognition and cleavage by Cas proteins due to the high mutation rates inherent to MGEs such as phages and plasmids^65,66^. Furthermore, MGEs can acquire anti-CRISPR (Acr) proteins, which directly inhibit various stages of the CRISPR-Cas pathway, effectively neutralizing the host’s defense^67,68^. The limited diversity of spacers within CRISPR arrays also presents a vulnerability, as MGEs with target sequences absent from the host’s array can evade recognition^69^. Finally, the requirement for a specific protospacer adjacent motif (PAM) for Cas protein activity introduces another constraint, as MGEs lacking the appropriate PAM can escape targeting^70^. These limitations highlight the ongoing evolutionary arms race between bacteria and their mobile genetic elements, demonstrating that while CRISPR-Cas systems provide a significant barrier against MGE invasion, they do not offer complete protection.

## Conclusion

In conclusion, three potential probiotic isolates out of five *Lactobacillus* isolates was screened for the probiotic properties. The promising samples were isolated from local dairy products (yogurt and sour milk) in Egypt, identified and confirmed as *L. delbrueckii* subsp. *bulgaricus* was related to its capacity to endure stressful situations, including NaCl, acid, bile salts, and the antimicrobial activity. If probiotic strains with antibiotic resistance are given along with antibiotic course, this might be advantageous. Studies were conducted to characterise the genomes and get a knowledge of the physiology, safety, and effectiveness of these isolates for probiotic properties using an in-silico method. The absence of virulence factors and pathogenicity in this study emphasizes its advantageous qualities, making it a viable option for use as a probiotic supplement. The flexibility of the genome is influenced by the existence of mobile genetic components and prophages rather than absence of antibiotic resistance genes. However, more in vivo studies on the animal and human clinical trials are required to accurately determine the safety, beneficial effects and efficacy of these promising candidates.

## Electronic supplementary material

Below is the link to the electronic supplementary material.

Supplementary Material 1

## Data Availability

The complete DNA sequence of the promising isolates was submitted to NCBI GenBank (submission number SUB14471673). The raw Sequencing Read Archives (SRA) was deposited in the NCBI under biosample accession numbers (B1: SAMN41534504, B2: SAMN41534505 and G1: SAMN41534506). This whole-genome sequence and all metadata for this bioproject was deposited at NCBI GenBank under (accession number PRJNA1116554).
