# Publisher Correction: Whole genome sequencing of promising *Lactobacillus delbrueckii* subsp. *bulgaricus* strains isolated from Egyptian dairy products for probiotic characteristics

**DOI:** 10.1038/s41598-025-97998-x

**Published:** 2025-04-23

**Authors:** Mostafa F. El-Hosseny, Mervat G. Hassan, M. O. Abdel-Monem, Mohammed G. Seadawy

**Affiliations:** 1 Botany and Microbiology Department, Faculty of Science, Banha University, Banha, Egypt; 2 Biodefense Center for Infectious and Emerging Diseases, Ministry of Defense, Cairo, Egypt

Correction to: *Scientific Reports* 10.1038/s41598-025-90262-2, published online 26 February 2025

The original version of this Article contained an error in the author list, where the name of the first author Mostafa F. El-Hosseny was inadvertently duplicated as Mostafa Fetoh Elhosseny and added as a last author. Mostafa F. El-Hosseny is the first and corresponding author, the duplicated name has been removed.

The original Article has been corrected.

