# Supplementary material for: Whole genome sequencing of promising Lactobacillus delbrueckii subsp. bulgaricus strains isolated from Egyptian dairy products for probiotic characteristics
Source: Sci Rep. 2025 Feb 26;15:6901. doi: 10.1038/s41598-025-90262-2 (PMC11865268; doi:10.1038/s41598-025-90262-2)
Supplement: Supplementary file 1 — Supplementary Material 1 [file 41598_2025_90262_MOESM1_ESM.docx]

**Abbreviations**

| LAB | Lactic acid bacteria |
| --- | --- |
| GRAS | Generally recognized as safe |
| FDA | The United States Food and Drug Administration |
| WGS | Whole genome sequence. |
| MRS | De man, rogosa and sharpe |
| CLSI | Chemical Laboratory Standard Institute |
| OD | Optical density |
| MHA | Mueller-Hinton agar |
| MTT | 3-(4,5-dimethylthiazol-2-yl)-2, 5-diphenyltetrazolium bromide |
| DMEM | Dulbecco’s Modified Eagle’s ‎Medium |
| PBS | Phosphate buffer saline |
| PFU | Plaque-forming unit |
| PATRIC | PathoSystems Resource Integration Center |
| RASTtk | Rapid Annotation using Subsystem Technology tool kit |
| NCBI | National Center for Biotechnology Information |
| PGFams | PATRIC global protein families |
| MUSCLE | Multiple sequence alignment |
| RaxML | Randomized Axelerated Maximum Likelihood |
| CRISPR | Clustered regularly interspaced short palindromic repeats |
| Cas | CRISPR associated-genes |
| CDS | protein coding sequences |
| tRNA | transfer Ribo-Nucleic Acid |
| rRNA | ribosomal Ribo-Nucleic Acid |
| EC | Enzyme Commission numbers |
| GIt | Gastro Intestinal Tract |
| MGEs | mobile genetic elements |
| PAM | protospacer adjacent motif |
